# Genomic Insights into Omega-3 Polyunsaturated Fatty Acid Producing *Shewanella* sp. N2AIL from Fish Gut

**DOI:** 10.3390/biology11050632

**Published:** 2022-04-21

**Authors:** Anchal Chaudhary, Omkar Avinash Ketkar, Sayed Irfan, Varnika Rana, Praveen Rahi, Rupesh Deshmukh, Jagdeep Kaur, Hena Dhar

**Affiliations:** 1 National Agri-Food Biotechnology Institute (NABI), Mohali 140306, India; anchal.chaudhary@nabi.res.in (A.C.); varnika.rana@nabi.res.in (V.R.); rupesh@nabi.res.in (R.D.); 2 Department of Biotechnology, Panjab University, Chandigarh 160025, India; jagsekhon@pu.ac.in; 3 National Centre for Microbial Resource, National Centre for Cell Science, Pune 411007, India; omkarketkar97@gmail.com (O.A.K.); sayedirfan631@gmail.com (S.I.); praveen@nccs.res.in (P.R.)

**Keywords:** *Shewanella*, genome sequencing, polyunsaturated fatty acids, secondary metabolites, stress resistance

## Abstract

**Simple Summary:**

The microbial production of health-beneficial omega-3 fatty acids has gained research attention due to the limitations of availability, sustainability, and contaminants associated with the conventional fish source. Among the marine bacteria, the *Shewanella* genus is well known for eicosapentaenoic acid (EPA) production along with bioremediation of organic and inorganic pollutants and bioelectricity generation. We report the genome sequence of an EPA-producing bacterium, *Shewanella* sp. N2AIL, isolated from the gut of a freshwater fish, Tilapia. Analysis of genomic data revealed a variety of antibiotic and stress-responsive genes, metal-reducing genes, and secondary metabolite genes. Overall, the study has provided an understanding of the biosynthetic gene cluster of omega-3 polyunsaturated fatty acids in addition to adaptation strategies of *Shewanella* sp. N2AIL and could be utilized in developing a strain for the commercial production of EPA.

**Abstract:**

The genus *Shewanella* is widely distributed in niches ranging from an aquatic environment to spoiled fish and is loaded with various ecologically and commercially important metabolites. Bacterial species under this genus find application in bioelectricity generation and bioremediation due to their capability to use pollutants as the terminal electron acceptor and could produce health-beneficial omega-3 fatty acids, particularly eicosapentaenoic acid (EPA). Here, the genome sequence of an EPA-producing bacterium, *Shewanella* sp. N2AIL, isolated from the gastrointestinal tract of Tilapia fish, is reported. The genome size of the strain was 4.8 Mb with a GC content of 46.3% containing 4385 protein-coding genes. Taxonogenomic analysis assigned this strain to the genus *Shewanella* on the basis of average nucleotide identity (ANI) and in silico DNA-DNA hybridization (DDH), phylogenetically most closely related with *S*. *baltica* NCTC 10735^T^. The comparative genome analysis with the type strain of *S*. *baltica* revealed 693 unique genes in the strain N2AIL, highlighting the variation at the strain level. The genes associated with stress adaptation, secondary metabolite production, antibiotic resistance, and metal reduction were identified in the genome suggesting the potential of the bacterium to be explored as an industrially important strain. PUFA synthase gene cluster of size ~20.5 kb comprising all the essential domains for EPA biosynthesis arranged in five ORFs was also identified in the strain N2AIL. The study provides genomic insights into the diverse genes of *Shewanella* sp. N2AIL, which is particularly involved in adaptation strategies and prospecting secondary metabolite potential, specifically the biosynthesis of omega-3 polyunsaturated fatty acids.

## 1. Introduction

The genus *Shewanella* was named after James Shewan, a Scottish bacteriologist known for his work in marine microbiology [1]. The first strain was identified as *Achromobacter putrefaciens* isolated from rancid butter by Derby and Hammer in 1931, which was further renamed *Pseudomonas putrefaciens* in 1941 and then *Alteromonas putrefaciens* in 1977 [2,3]. In 1985, MacDonell and Colwell finally reclassified it into *Shewanella* and identified it as Gram-negative, motile, rod-shaped, facultatively anaerobic bacteria [4]. It belongs to the class Gammaproteobacteria and order *Alteromonadales* distributed in diverse habitats such as freshwater lakes, ocean sediments, deep sea, iced sea, oily fields, hydrothermal vents, and intertidal warm water, and also from the intestine of fishes and marine invertebrates [5,6,7,8,9,10,11]. The important characteristic feature of *Shewanella*, which allow it to grow in a diverse range of niches, is its capacity to reduce various organic and inorganic compounds for anaerobic respiration. *Shewanella* is characterized by its ability to employ an external electron transfer system to transfer electrons from the quinone pool to the outer membrane of a cell by c-type cytochromes assembly [12]. Metals such as iron, manganese, and mercury have been shown to be reduced by *Shewanella* [13,14]. As a result of this property, *Shewanella* spp. has drawn attention in the field of bioremediation for the degradation of hydrocarbons, dyes, various pollutants, and pesticides [15,16,17]. The biological metal reduction potential of *Shewanella* allows it to reduce electrodes and results in bio-electricity generation [18]. *Shewanella* genus show versatility in response to common physiological conditions such as temperature and salt concentration. Some species are psychrotrophic, some are mesophilic, and the genus also contains species that can tolerate extremely high temperatures above 40 °C. Most of the strains of this genus are moderate halophiles but some isolates show high growth rate even in the presence of high salt concentration [19]. In response to high salinity, various transport systems have been employed by *Shewanella* for the exit of harmful ions [20]. The species of this genus also show diversity in response to fluctuating pH and adopted various resistance mechanisms in order to respond to environmental conditions such as low temperature by expressing cold shock expression genes [21]. 

*Shewanella* is also known to be the key species in the area of the food industry as a food spoilage organism. *Shewanella baltica*, being a psychrotrophic bacteria, was known for the spoilage of fish food products [22]. Recently, alginate lyase purified from *Shewanella* sp. YH1 has been used in the food industry as a gelling agent and stabilizer [23]. Apart from these, the most important feature of this genus is its ability to produce omega-3 fatty acids, particularly eicosapentaenoic acid (EPA), which is an important resistant mechanism adopted by *Shewanella* to thrive in low temperature, saline, and high-pressure conditions by changing lipid composition to maintain membrane fluidity [24,25,26]. The strains of *Shewanella*, including *S*. *violacea*, *S*. *benthica*, *S*. *peizotolerans*, have been reported to produce EPA, which is associated with adaptation under extreme cold and high hydrostatic pressure [27,28,29]. These omega-3 fatty acids are associated with beneficial health effects related to maternal and fetal health, prevention of cardiovascular diseases, preventing blood circulatory disorders due to the anti-aggregatory feature of EPA, and prevention of neurological disorders [30,31,32,33]. Due to the above salient features, the *Shewanella* genus has been of research interest in environmental and applied microbiology. The diversity in habitat and in response to external environmental conditions led to increased interest in the genome analysis of *Shewanella* to explore some interesting features. The steady increase in the availability of *Shewanella* genomes sequences brought the need for annotation of genome data for uncovering genes of paramount importance. Furthermore, *Shewanella* spp. has served as a model for evolutionary studies at the genome level due to the correlation of evolutionary likeness with genotypic and phenotypic similarities among the organisms [34]. 

In this study, we report the genome sequence of an EPA-producing bacterium, *Shewanella* sp. N2AIL, to identify the diverse biosynthetic gene clusters with major emphasis on PUFA production. Furthermore, the comparative genome analysis of *Shewanella* sp. N2AIL with closely related type strains was performed to gather insights into its evolutionary relatedness. The genes linked with important stress responses such as temperature, salinity, acid and alkaline stress, osmotic stress, and heavy metal stress were also explored to understand the survival strategies adopted by *Shewanella* sp. N2AIL to cope with external stresses.

## 2. Materials and Methods 

### 2.1. Identification and Phylogenetic Analysis of Bacterial Strain

The bacterial strain N2AIL isolated from gastrointestinal tract of fish was revived from glycerol stock maintained at −80 °C on Luria-Bertani (LB) agar at 28 °C for 24 h. The strain was cultivated in LB broth for 16 h at 28 °C to be used as inoculum. Genomic DNA was extracted from overnight grown culture using the Wizard^®^ Genomic DNA Purification Kit (Promega, Madison, Wisconsin, USA) based on manufacturer’s guidelines. A 16S rRNA gene was amplified using the universal primers 27f (5′-AGAGTTTGATCCTGGCTCAG-3′) and 1492r (5′-TACGGYTACCTTGTTACGACT-3′) [35,36] and subjected to 16S rRNA gene sequencing by employing Big Dye^®^ Terminator v3.1 Cycle Sequencing Kit (Applied BiosystemsTM, Foster City, CA, USA) [37]. The 16S rRNA gene sequence of N2AIL was compared with that of closely related species downloaded from NCBI database by performing phylogenetic analysis in MEGA11 (Molecular Evolutionary Genetics Analysis) software using maximum likelihood method with 1000 bootstrap [38]. 

### 2.2. Genome Sequencing and Its Assembly

Quality of the genomic DNA of N2AIL strain extracted using the Wizard^®^ Genomic DNA Purification Kit (Promega, Madison, WI, USA) was assessed using nanodrop lite spectrophotometer (Thermofisher, Wilmington, DE, USA) and Qubit dsDNA HS assay kit. The library was prepared using Nextera DNA Flex Library preparation kit (Illumina), and the quality and quantity of library were checked using the Qubit dsDNA HS assay kit and Agilent 2200 Tapestation. The library was sequenced using Illumina Miseq platform with 2 × 250 bp v2 chemistry platform (Illumina Inc., San Diego, CA, USA). Sequencing data were then assembled de novo using SPAdes version 3.10.0 (Saint Petersburg, Russia). The raw data quality assessment was performed using FastQC-Toolkit v0.11 (Babraham, UK), and the low-quality reads were filtered using NGS QC-Toolkit v2.3.3 (New Delhi, India).

### 2.3. Taxonogenomic Analysis

The genomic relatedness of strain N2AIL with the closely related type strains of the genus *Shewanella* was measured based on average nucleotide identity (ANI) and digital DNA-DNA hybridization (dDDH) values. Pairwise ANI value between genome sequences was calculated by OrthoANIu algorithm based EZ BioCloud ANI Calculator tool [39]. The in silico DDH value was calculated by Genome-to-Genome distance calculator (GGDC) version 3.0 for comparison of genomes. The ANI and DDH matrix and heat map among the genomes were generated using Orthologous Average Nucleotide Identity Tool (OAT) v 0.93.1 tool (Chunlab Inc., Seoul, Korea). Whole-genome phylogenetic tree was inferred by PhyML based REALPHY (Reference sequence alignment based phylogeny builder) tool version 1.13 where the provided query and reference sequences were mapped using Bowtie 2 tool [40]. 

### 2.4. Genome Annotation

RAST (Rapid Annotation using Subsystem Technology) v 2.0 tool was used for rapid annotation of genome by identifying protein-encoding genes and their functions, tRNA and rRNA genes. The tRNA and rRNA encoding genes were identified in RAST by tRNAscan-SE and “search_for_rnas” tools, respectively [41]. Clustered, regularly interspaced short palindromic repeats (CRISPRs) were determined by CRISPR Finder tool [42]. Antibiotic-resistant genes in genome were predicted through CARD (Comprehensive Antibiotic Resistance Database) using RGI (Resistance Gene identifier) based on protein homology modeling and SNP model [43]. Secondary metabolite gene clusters were predicted by the antiSMASH 5.0 (antibiotics and Secondary Metabolite Analysis Shell) online tool, which covers wide range of secondary metabolite classes [44]. Functional categorization of genes into cluster of orthologous classes was obtained on WebMGA tool using RPSBLAST program against COGs (Clusters of Orthologous Groups of proteins) databases using default *E*-*value* settings [45]. The pictorial representation of *Shewanella* sp. N2AIL genome was drawn using GView server (https://server.gview.ca/ (accessed on 7 March 2022)).

### 2.5. Comparative Genomic Analysis

The closely related type strains of *Shewanella* sp. N2AIL was selected for comparative genomic analysis. Genome comparison, including genome rearrangements and deletions, was performed between *Shewanella* sp. N2AIL and *S*. *baltica* NCTC 10735^T^ using MAUVE tool with default parameters [46]. The genomes of closely related type strains were annotated using the Prokka Prokaryotic genome annotation (Galaxy 1.14.5) tool [47]. The comparison of shared and unique genes among them was performed using EVenn tool with default parameters [48]. 

### 2.6. Domain Prediction and 3D Structure Modelling

SMART-EMBL (Simple modular architecture research tool) and InterProScan tool were used to predict the overall domain architecture and extraction of significant protein domain sequences [49]. The 3D structure of different domains was predicted by Phyre2 tool [50]. Subsequent quality check and structure validation were performed with ProTSAV (Protein Structure analysis and validation) [51] and SAVES6.0 (https://saves.mbi.ucla.edu/ (accessed on 13 January 2022)) tools. The stability of protein structure was analyzed on the basis of RMSD (root mean square deviation) value calculated by aligning the individual domain model with its closest PDB model using PyMOL (https://pymol.org/2/ (accessed on 13 January 2022)). The crystal structures used as templates for pfaA protein were—bacillaene polyketide synthase of *Bacillus amyloliquifaciens* (PDB accession- 6MHK) for KS domain, MCTA (malonyl CoA acyl carrier protein transacylase) from *Streptococcus pneumonia* (PDB accession- 3IMK) for AT domain, acyl carrier protein from *Thermus thermophilus* Hb8 (PDB accession-1X3O_A) for ACP domain, Ketoreductase domain from bacillaene assembly line of *Bacillus subtilis* 168 (PDB accession- 4J1Q_A) for KR domain and Curacin polyketide synthase CurF module (PDB accession- 3KG6) for DH domain. For AT domain of pfaB protein, the template was acyltransferase domain of salinomycin acyl transferase (PDB accession- c6iyO_A). For pfaC protein, the template for KS and CLF was KS-CLF didomain of LC-PUFA synthase of *Moritella marina* (PDB accession- 6RIW), and for DH domains, the template was 3-hydroxy decanoyl (ACP) DH from *Yersinia pestis* (PDB accession- 3Q62), and for pseudo-DH domain, DH domain of mammalian fatty acyl synthase (PDB accession- c2cf2L). The structural template for Enoyl reductase domain of PfaD was Enoyl reductase from trans-AT polyketide synthase (PDB accession- 4YX6_A). The *pfaE* domain structural template selected was phosphopantetheine transferase Peptidyl carrier protein (PDB accession- 4MRT_A).

### 2.7. Data Availability

The draft genome sequence and 16S rRNA gene sequence of *Shewanella* sp. N2AIL were submitted to NCBI with accession numbers JALBYT000000000 and OM919690, respectively. The version described in this paper is version JALBYT010000000.

## 3. Results and Discussion

### 3.1. Genome Structural Features

**Figure 1:**
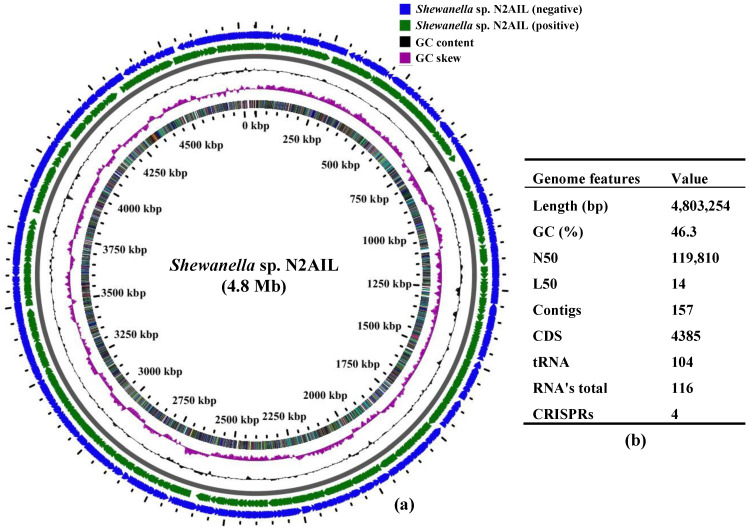
Circular genome map of *Shewanella* sp. N2AIL. Circles from the outside to inside showing: (1) Antisense strand (2) Sense strand (3) GC content and (4) GC skew (**a**), and table showing its general genome features (**b**).

The final assembled *Shewanella* sp. N2AIL genome consisted of 157 contigs with a total genome size of 4,803,254 bp and a mean G + C content of 46.3% based on RAST annotation (Figure 1). The N50 size and L50 size were equal to 119,810 bp and 14 bp, respectively. The size difference between the draft genome sequence of the strain N2AIL and the complete genome sequence of its closest related strain, *S*. *baltica* NCTC 10735, was around 0.5 Mb, while the GC content is comparable (Supplementary Table S1). A total of 4385 protein-coding sequences (CDSs) and 116 total RNA were found in the genome (Figure 1). Out of them, 104 were tRNAs, and 12 were rRNAs. Total CRISPR elements contained in *Shewanella* sp. N2AIL genome were four in number (Supplementary Table S2), equal to the number present in the genome of its closest strain, *S*. *baltica* NCTC 10735.

### 3.2. Phylogenetic and Taxonogenomic Description 

**Figure 2:**
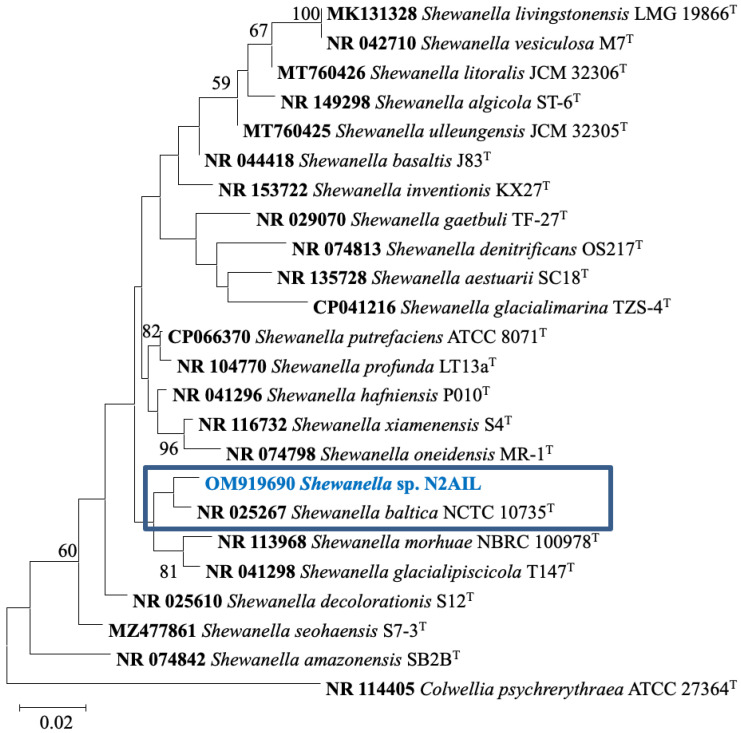
Maximum likelihood phylogenetic tree based on the 16S rRNA gene sequences of *Shewanella* sp. N2AIL along with related type strains. The values on the tree indicate the bootstrap percentages obtained after 1000 replications. The type species *Colwellia psychrerythraea* ATCC 27364^T^ was used as an outgroup. Scale bar represent 2% genetic variation.

**Figure 3:**
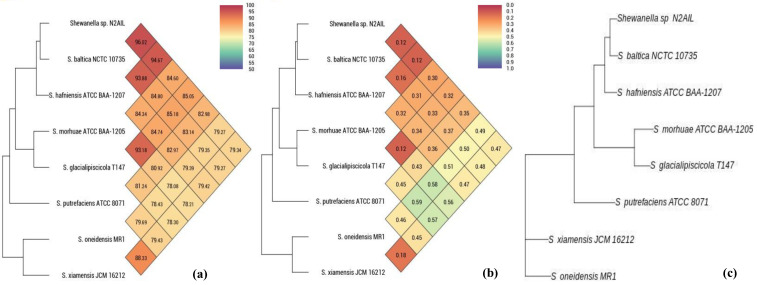
Heat map and phylogenetic tree based on ANI (**a**), GGDC distance (**b**), and whole-genome sequence of *Shewanella* sp. N2AIL (**c**) with its closely related taxa.

The taxonomic status of *Shewanella* sp. N2AIL was confirmed by BLAST-search of 16S rRNA gene sequence of length 1454 bp. The most closely related strains were *S*. *baltica* NCTC 10735 followed by *S*. *hafniensis* ATCC BAA-1207 with a similarity score of 98.55% and 97.38%, respectively. A maximum-likelihood tree showed the clustering of *Shewanella* sp. N2AIL with *S*. *baltica* NCTC 10735 (Figure 2). Genome level similarity assessed based on ANI score showed a maximum of 96.02% ANI value of *Shewanella* sp. N2AIL with *S*. *baltica* strain NCTC10735, which is slightly higher than the speciation threshold (95–96%) recommended for a strain to be considered as belonging to the same species [52]. The ANI value was below 95% with the other reference genomes (Supplementary Table S1). On the contrary, in silico DDH analysis showed the DDH score of 66.5% for *Shewanella* sp. N2AIL with *S*. *baltica* strain NCTC10735 followed by 58.2% with *S*. *hafniensis* ATCC BAA-1207, which is lower than the threshold (70%) recommended for species delineation (Supplementary Table S2) [53]. Both the ANI and dDDH analysis together could not form the basis for species demarcation as per the threshold recommended for a strain belonging to novel species. The dendrogram constructed based on ANI and dDDH values showed that the *Shewanella* sp. N2AIL was found to be more closely related to *S*. *baltica* NCTC 10735 with an ANI value of 96.02% and GGDC distance of 0.12, followed by *S*. *hafniensis* ATCC BAA-1207 with an ANI value of 93.88% and GGDC distance of 0.16 and *S*. *glacialipiscicola* T147 with an ANI value of 93.18% and GGDC distance of 0.12 (Figure 3a,b). The whole-genome phylogeny constructed using the REALPHY tool confirmed the closeness of *Shewanella* sp. N2AIL with *S*. *baltica* NCTC 10735 followed by *S. hafniensis* ATCC BAA-1207 (Figure 3c) and is similar to phylogeny construction based on ANI and dDDH values. 

### 3.3. Comparative Genome Analysis 

**Figure 4:**
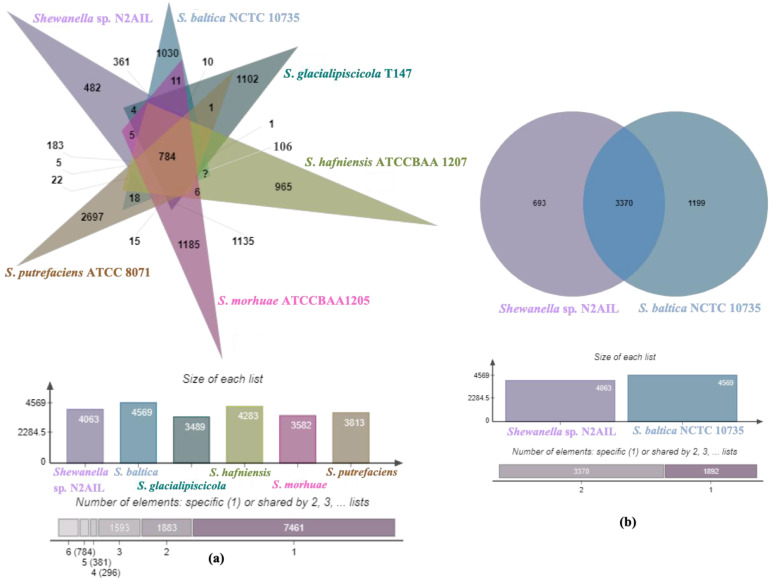
Venn diagram showing the distribution of shared and unique gene clusters of *Shewanella* sp. N2AIL with the related taxa (**a**), and comparison of the genes with the most closely related strain *S*. *baltica* NCTC 10735 (**b**).

**Figure 5:**
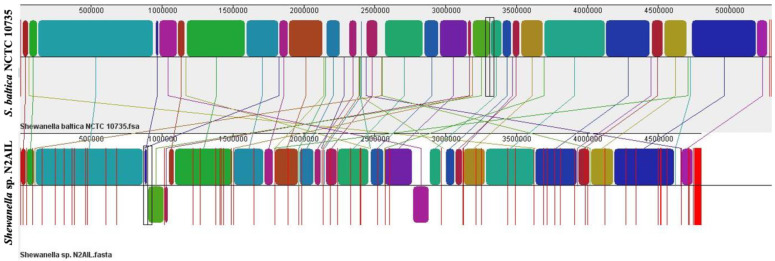
Genome alignment of *Shewanella* sp. N2AIL and *S*. *baltica* NCTC 10735 performed using MAUVE showed variation in genome structure. The scale shown above represents coordinates of each genome. LCB’s (Local Collinear Blocks) shown in different colours represented conserved segments in both the genomes. White area present within the LCB’s represents the regions with low similarity. LCBs shown above the central black horizontal line are in forward orientation and below are in reverse orientation. Coloured lines show rearrangements between two genomes.

The genome comparison of closely related type strains of *Shewanella* sp.N2AIL is shown in Supplementary Table S1. There was a difference in genome size ranging from 4.1 Mb to 5.3 Mb and GC content from 41.4 to 46.3% among these type strains. To characterize the genomic composition of the *Shewanella* sp. N2AIL, genomes of the closely related type strains were selected for pan-genome analysis. *Shewanella* sp. N2AIL exhibited a pan-genome size harboring 12,398 genes. Out of them, only 784 were identified as core genes, and 11614 genes were identified as shell genes. The core and variable gene pools among six reference strains are shown in Figure 4. The unique gene pool constitutes 73.6% of the total gene pool of the six studied genomes, and their number varies from 482 in *Shewanella* sp. N2AIL to 965 in *S*. *hafniensis* ATCC BAA1207, 1030 in *S*. *baltica* NCTC 10735, 1102 in *S*. *glacialipiscicola* T147, 1185 in *S*. *morhuae* ATCC BAA1205, and 2697 in *S*. *putrefaciens* ATCC 8071 (Figure 4a). Among these clusters, 381, 296, 1593, 1883 and 7461 genes were conserved in the 5, 4, 3, 2, and 1 genome, respectively (Figure 4a). Further, a comparison of *Shewanella* sp. N2AIL with the most closely related species *S*. *baltica* NCTC 10735^T^ revealed sharing of 3370 core homologous gene clusters, constituting 64.04% of the total gene pool, and specific gene clusters were 693 and 1199, respectively (Figure 4b). Comparative genome analysis using Mauve was carried out to understand genome-wide dynamics of *Shewanella* sp. N2AIL and *S*. *baltica* NCTC 10735. The genomes displayed great variation in their structure, as seen by numerous rearrangements and inversions (Figure 5). Comparison between both the genomes showed that there were 47 LCB’s (Local Collinear Blocks) with a maximum LCB weight of 445898 and a minimum of 73. The minimum LCB weight could be due to the draft genome of *Shewanella* sp. N2AIL compared with complete genome of *S*. *baltica* NCTC 10735. In addition, there were some blank regions in two genomes that represent strain-specific features. 

### 3.4. Genome Functional Annotation

**Figure 6:**
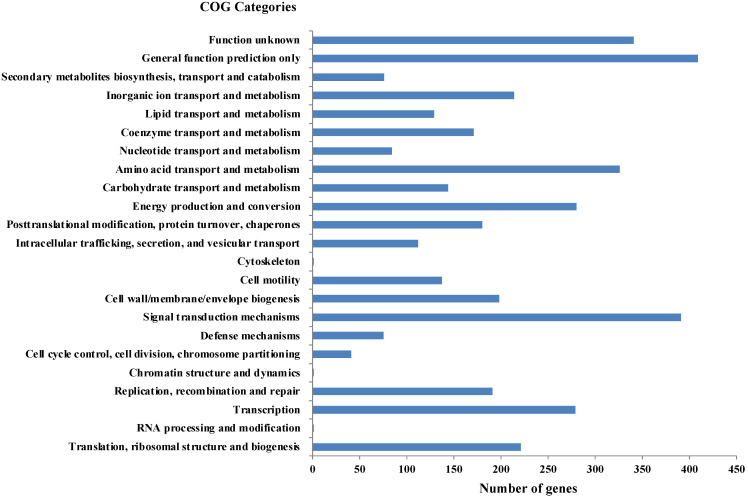
Distribution of COG functional categories of *Shewanella* sp. N2AIL genome.

As per RAST annotation, 418 for biomolecule metabolism, 156 for Membrane transport, 74 for stress response, and 69 for element metabolism, and 48 genes encoding for bacterial resistance were identified in *Shewanella* sp. N2AIL genome (Supplementary Figure S1). Among the genes involved in biomolecule metabolism, a total of 189 genes were identified for carbohydrate metabolism, 227 for protein metabolism, 58 for fatty acid and lipid metabolism, and 144 for nucleic acid metabolism. Genes associated with membrane transport, including ABC transporters, cation transporters, and TRAP transporter, were identified. The stress-responsive genes include genes for osmotic stress, oxidative stress, and periplasmic stress. Out of 69 genes encoding metabolism of important elements, 9 were for potassium metabolism, 12 for iron metabolism, 21 for sulfur, and 27 for phosphorus assimilation, and also 48 genes involved in disease, virulence, and defense were also reported in *Shewanella* sp. N2AIL genome. Further, protein-coding genes obtained from WebMGA were found to be clustered into orthologous classes. The distribution of genes into COGs functional categories (Clusters of Orthologous Groups of proteins) is shown in Figure 6. A major percentage of genes fall in the category of General function prediction (10%), followed by Signal transduction (9.8%) and amino acid transport and metabolism (8.1%) of total genes. A large number of genes also fall into the unknown function category (8.5%). The relative distribution of genes into different functional categories such as E: amino acid metabolism (326 genes), C: energy production and conversion (280), J: translation, ribosomal structure, and biogenesis (221), L: Replication, recombination, and repair (191), K: Transcription (279), G: Carbohydrate metabolism (144), Signal transduction (391), secondary metabolite synthesis (76), P: inorganic ion metabolism (214), General function prediction (409), function unknown (341), etc., was observed. A similar relative percentage of genes was also observed in the genome of *S*. *baltica* 128, accounting for 9.85% and 8.66% for signal transduction and amino acid metabolism, respectively [54]. Similarly, *S*. *algae* ACCC accounts for about 5.35% of genes for signal transduction, followed by 5.49% for translation, ribosomal structure, and biogenesis [55], and *S*. *peizotolerans* WP3 has a slight increase in cell wall formation and energy production [28]. Altogether, it was indicated that the signal transduction and amino acid metabolism genes are majorly present in the *Shewanella* sp. N2AIL genome, similar to *S*. *baltica* 128. 

### 3.5. Prediction of Secondary Metabolite Gene Clusters 

*Shewanella* sp. N2AIL genome includes secondary metabolite gene clusters such as betalactone, PUFA, siderophore, RiPP (post-translationally modified peptide), and arylpolene based on antiSMASH 5.0 tool analysis (Table 1). The total fraction of genes encoded in these clusters include PUFA (40 genes), RiPP (8 genes each), siderophore (9 genes), betalactone (22 genes), and arylpolyene (27 genes). The biosynthetic genes found in these clusters comprise mainly of core biosynthetic genes, additional biosynthetic genes, and transport and regulatory genes. Similarly, these five secondary metabolite gene clusters were also reported in the *Shewanella* sp. strain Lzh-2 genome sequence [56]. Furthermore, similar types of gene clusters were analyzed in the closest relative strain of *Shewanella* sp. N2AIL, i.e., *S*. *baltica* NCTC 10735. The biosynthetic gene cluster of arylpolyene was reported to be the largest and most prominent family of biosynthetic gene clusters, which have a protective role in oxidative stress tolerance [57]. The widespread presence of another biologically active antimicrobial agent, RiPP, was reported in anaerobic bacteria, including Actinobacteria, Proteobacteria, and Firmicutes by genome mining [58]. The Polyketide synthase gene cluster is known to synthesize antibiotics having pharmaceutical value [59]. The presence of such useful biosynthetic gene clusters in *Shewanella* sp. N2AIL enable it to adapt to extreme environments and have been exploited for pharmaceutical and industrial importance. 

### 3.6. Antibiotic Resistant Gene Prediction

Antibiotic-resistant gene annotation based on CARD tool revealed the presence of antibiotic-resistant genes for fluoroquinolone, tetracycline, diaminopyrimidine, phenicol, elfamycin, and Carbapenem, Cephalosporin, and Penam antibiotic classes in *Shewanella* sp. N2AIL (Table 2). Similarly, all these antibiotic-resistant genes were also identified in closely related strain *S*. *baltica* NCTC 10735. In contrast, multidrug-resistant genes for colistin, imipenem, ampicillin, and cefazolin antibiotics were identified in *Shewanella algae* YHL [60]. It was observed that the antibiotic resistance in bacteria could not be explained solely at the genome level; phenotypic studies are needed to confirm the antibiotic resistance. A spoilage bacteria, *Dermacoccus abyssi*, showed resistance to fluoroquinolone, aminoglycosides, and tetracycline antibiotics at the genome level but showed susceptibility to the same phenotypically [61].

### 3.7. Adaptation to Stress

The genome of *Shewanella* sp. N2AIL contains numerous genes which have been reported to be associated with stress adaptation, such as temperature, salt, oxidative, pH, osmotic stress, and heavy metal stress (Table 3). In order to combat temperature stress, bacteria generally employ cold shock (Csp) and heat shock proteins (Hsp) that serve as RNA chaperones and are important in protein folding and transportation, respectively. Four Csp and six Hsp were identified in the *Shewanella* N2AIL genome. The expression of two cold shock proteins, CspA and CspG, was reported to be upregulated in *S. violacea* strain DSS12 on exposure to low temperature [62]. Further, the *rpoE* gene responsible for bacterial growth at low and high temperatures, high salinity and nutrients scarcity, and RpoE dependant serine protease DegQ associated with growth at high temperatures were also predicted in *Shewanella* sp. N2AIL genome. The presence of these *rpoE* and *degQ* genes in *S*. *oneidensis* MR-1 were reported to affect temperature stress resistance [63], indicating their role in temperature stress adaptation in *Shewanella* sp. N2AIL. 

The genes *envZ* and *ompR* encoding a bacterial two-component stress sensor proteins were found in *Shewanella* sp. N2AIL genome for imparting osmotic stress tolerance. The functional assessment of these genes based on mutational analysis was performed in *S*. *oneidensis* for evaluating its role in osmoregulation [64]. Furthermore, genes such as *kdpA*, *kdpB*, *trkH* encoding potassium transporter system and *betA*, *betB* encoding betaine and choline transporters responsible for mediating salt stress response were identified in *Shewanella* sp. N2AIL genome. This strategy for salt stress resistance was employed by *S. algae* 2736 by upregulating betaine and choline transporters for the accumulation of solutes such as betaine and choline in response to high salt concentration [65]. 

Additionally, genes related to pH stress response, such as acid and alkaline stress, were identified in the *Shewanella* sp. N2AIL genome (Table 3). Acid-stress-resistant genes *pstA*, *pstB* encoding phosphate ABC transporters and *PhoB*, *PhoU* encoding phosphate regulatory proteins, and *csgBEFG* for cell envelope structure were identified in *Shewanella* sp. N2AIL genome. The alkaline-stress-resistant genes *nhaA*, *nhaR* related to the Na^+^/H^+^ antiporter system, and *cysPTWAB* encoding Sulfate ABC transporter proteins were also identified in *Shewanella* sp. N2AIL. The elevated expression of these acid and alkaline stress-responsive genes was obtained in *S*. *oneidensis* [66], suggesting the potential of *Shewanella* sp. N2AIL against pH stress response.

Major biotechnological application of *Shewanella* is associated with bioremediation due to its extracellular metal reduction potential for metals such as iron, manganese, vanadium, palladium, mercury, platinum, uranium, and plutonium [12,13]. Heavy-metal-stress-responsive genes in response to major stresses such as copper, nitrate, selenite, iodine, and iron were identified in *Shewanella* sp. N2AIL. The genes *copA* and *cusA* encoding two important proteins for copper efflux, i.e., CpX Type ATPase and RND protein, were identified in *Shewanella* sp. N2AIL genome. These two proteins, CopA and CusA, were reported to have enhanced expression in *S*. *oneidensis* MR1 in response to copper challenge [67], suggesting the role of CopA and CusA proteins in copper resistance. Furthermore, nitrate-reducing genes *nap* and its response regulator *nar* were identified in *Shewanella* sp. N2AIL genome. NarQ-P is a two-component system responsible for the regulation of nitrate-reducing gene- *nap*. These two nitrate reducing genes were expressed in *S*. *peizotolerans* WP3 for nitrate reduction under high hydrostatic pressure [68]. Selenite reducing genes such as *fccA* encoding periplasmic fumarate reductase and *cymA* encoding c-type cytochrome for supplying electrons to *fccA* were identified in *Shewanella* sp. N2AIL genome. The contribution of these genes in selenite reduction was previously evaluated for *S*. *oneidensis* MR-1 by mutational analysis [69] also indicates their role in selenite reduction in *Shewanella* sp. N2AIL genome.

**Figure 7:**
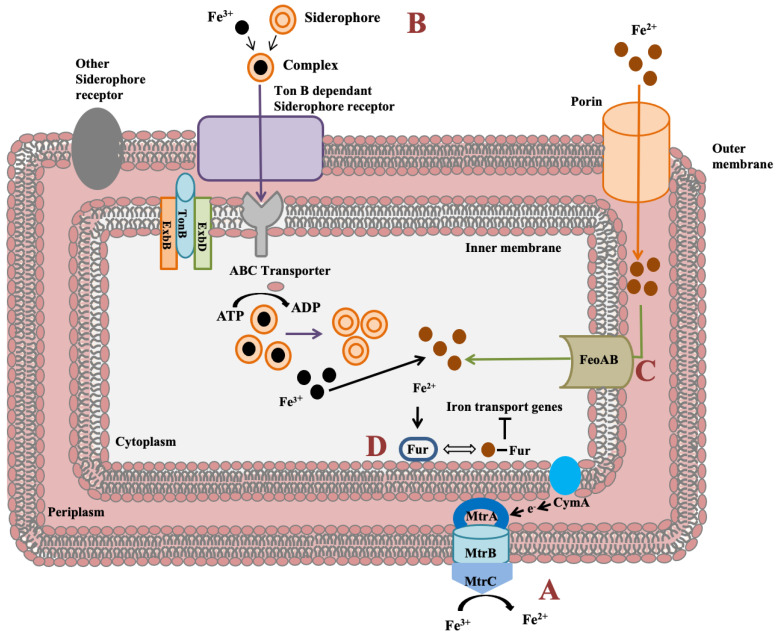
Iron uptake genes in *Shewanella* sp. N2AIL and their route. (A) The c-type cytochrome encoded by *mtrABC* cluster spanning outer membrane responsible for reduction of soluble ferrous form (Fe^2+^) to insoluble ferric form (Fe^3+^). (B) Fe^3+^ in the environment can be reduced to Fe^2+^ by siderophore-mediated iron uptake system via Ton B dependant receptor of *Shewanella* sp. N2AIL for uptake of Fe^3+^-Siderophore complexes and transported by ABC transporter to cytoplasm where Fe^3+^ is reduced to Fe^2+^ and released from the complex. (C) Another strategy employed is FeoAB system, a ferrous iron transporter for uptake of Fe^2+^ across inner membrane. (D) A global ferric uptake regulator encoded by *fur* gene control bacterial iron homeostasis.

*Shewanella* genus has an impressive feature to utilize wide range of compounds for iron reduction associated with biofilm formation and for energy generation. Notably, for iron reduction, the *mtrBAC-mtrFED* gene cluster encoding outer membrane cytochrome C was identified in *Shewanella* sp. N2AIL genome (Figure 7a). Shi et al. (2006) reported the role of the *omcA-mtrC* complex in an iron reduction in *S*. *oneidensis* MR-1 [70]. Further, the gene cluster reported for iron uptake in *S*. *oneidensis* was mtrC [71]. Furthermore, the role of primary gene *mtrC* and auxillary gene *undA* for iron reduction was indicated in *S*. *putrefaciens* W3-18-1 by mutational analysis [72]. Siderophore mediated iron uptake system for transport of insoluble form of iron-Fe^3+^ mediated by TonB dependant siderophore receptor and the TonB-ExbB-ExbD ferric siderophore transport system was identified in *Shewanella* sp. N2AIL genome (Figure 7b). Similarly, the TonB receptor PutA was reported in *S*. *oneidensis* for siderophore dependant iron uptake [73]. In addition to siderophore dependant route for iron uptake, a Feo system encoded by *feoA* and *feoB* genes was also identified in *Shewanella* sp. N2AIL for direct uptake of a soluble form of iron-Fe^2+^ (Figure 7c). The physiological role of Feo as a primary iron uptake protein was also demonstrated in *S*. *oneidensis* [74]. Further, a ferric uptake regulator protein encoded by the *fur* gene for maintaining iron homeostasis was also identified in *Shewanella* sp. N2AIL genome (Figure 7d). The role of Fur genes was investigated in *S*. *peizotolerans* WP3 by generating fur mutant under anaerobic growth conditions, and severe growth defect was observed in the mutant strain [75]. In addition, the presence of the *bolA* gene in *Shewanella* sp. N2AIL indicated its potential for bioelectricity generation as the BolA transcription factor is responsible for biofilm formation and generating electric current. Initially, the *bolA* gene in *E*. *coli* was reported as a transcription regulator for maintaining cell shape [76]. Later, it was demonstrated that BolA acts as a transcriptional switch in *E*. *coli* to transit from the cell proliferation stage to the attachment stage under harsh stress conditions and *bolA* overexpression favors biofilm formation in *E*. *coli* [77]. Similarly, the overexpression of *bolA* gene in *S*. *oneidensis* MR-1 demonstrated increased biofilm generation and current generation [78]. In bioelectrochemical system (BES), only *S*. *oneidensis* and *Geobacter sulfurreducens* were reported so far to be electroactive organisms. The presence of *bolA* gene in *Shewanella* sp. N2AIL opens the way for its regulation and optimization to generate electric current.

### 3.8. PUFA Biosynthetic Gene Cluster 

**Figure 8:**
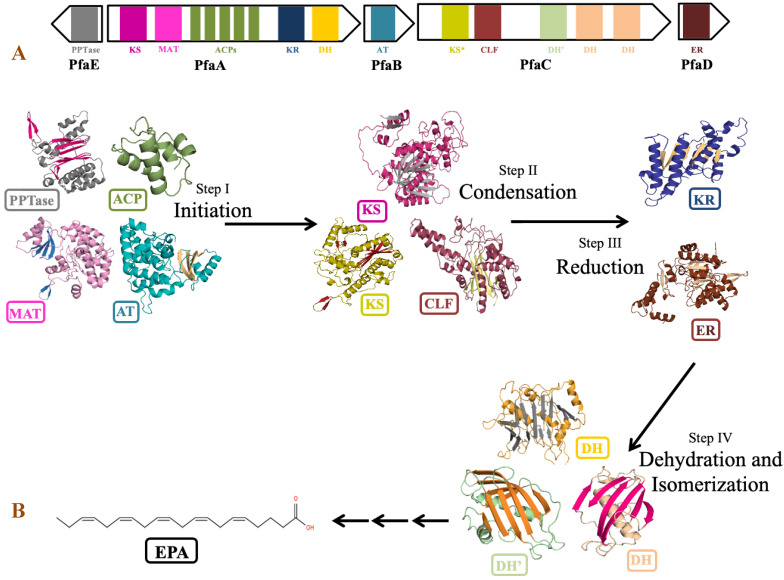
Domain organization and role of catalytic domains of EPA biosynthetic gene cluster of *Shewanella* sp. N2AIL in PUFA synthesis. (**A**) The catalytic domains are organized in five open reading frames (ORFs) represented in the form of arrows and the domains are represented in the form of differently colored solid blocks. (**B**) The major steps in EPA biosynthesis include: (i) initiation involving activation of acyl carrier proteins (ACP) by phosphopantetheine transferase (PPTase) which carries acyl and malonyl substrates transferred by acyltransferase (AT) and malonyl CoA:ACP acyl transferase (MAT) domains for fatty acyl chain elongation, followed by several cycles of (ii) condensation involving ketoacyl synthase (KS) and chain length factor (CLF) domains, (iii) reduction by ketoacyl-ACP reductase (KR) domain and (iv) dehydration by dehydratase (DH) domain and FabA-type dehydratase. Finally, elongation process is completed by reduction of double bond by enoyl reductase (ER).

The genus *Shewanella* is known to produce a wide range of secondary metabolites. Out of them, PUFA biosynthetic gene cluster is of major importance for the synthesis of biologically important compounds EPA and DHA. Detailed analysis of PUFA biosynthetic gene cluster predicted using antiSMASH revealed the presence of five genes *PfaA*, *PfaB*, *PfaC*, *PfaD*, and *PfaE* in its gene cluster as observed in the *pfa* gene cluster of Class marine Gammaproteobacteria including genus *Shewanella*, *Moritella*, *Colwellia*, *Photobacterium* and *Pseudoalteromonas* [79]. Four genes coding for *pfaA*, *pfaB*, *pfaC*, and *pfaD* proteins are present in one cluster, while *pfaE* is found outside the cluster in the antisense strand (Figure 8). Domain analysis by SMART-EMBL tool predicted the presence of functional enzymatic domains such as condensing domains: ketoacyl synthase (KS) and chain length factor (CLF); substrate selecting domains: acyltransferase (AT) and malonyl acyltransferase (MAT); carrier domain: acyl carrier proteins (ACPs); modifier domains: ketoacyl reductase (KR), enoylreductase (ER) and dehydratase (DH) in PUFA synthase gene cluster of *Shewanella* sp. N2AIL. These catalytic domains work in a specific sequential manner to carry out the overall process of fatty acid synthesis (Figure 8). Domain analysis of *PfaA* showed the presence of KS, MAT, five PP (Phosphopantetheine) binding ACP domains, and KR and DH domains. A similar arrangement of functional domains of PfaA was found in class Gammaproteobacteria with a variation in the number of ACP’s of *PfaA* gene. It was suggested that the number of ACPs plays an important role in regulating overall PUFA titer [80]. Further, it was suggested that apart from the number of ACPs, the structure of the ACP domain is also important for controlling PUFA production [81]. *PfaB* gene of *Shewanella* sp. N2AIL showed the presence of one AT domain. In *pfaB*, an additional KS domain is present in DHA-producing gene clusters but absent in EPA-producing ones [82]. It was reported that the KS domain of pfaB plays a central role in defining the final PUFA product [83]. The marked absence of the KS domain in *Shewanella* sp. N2AIL suggests its potential for EPA production only. The *pfaC* gene cluster of *Shewanella* sp. N2AIL is composed of heterodimeric KS-CLF didomain, FabA such as DH, and a pseudo-DH domain. The chain length factor is essential for chain elongation in very-long-chain omega-3 fatty acids, such as EPA and DHA. The disruption of the CLF domain of *Schizochytrium* decreases the DHA proportion without effect on EPA content [84]. Therefore, it was suggested that the presence of CLF is required for final elongation from C20 to C22, controlling the chain length. Further, the combination of both the DH domains and pseudo DH domains contributed to the functional activity of the DH domain [85]. The presence of both the domains in *Shewanella* sp. N2AIL suggested the complete functional potential of the DH domain. Domain analysis of PfaD showed the presence of nitronate monooxygenase (NMO) domain of enoyl reductase (ER). Further, the *pfaE* gene is located at a separate locus in *Shewanella* sp. N2AIL. PfaE protein has a PPTase domain (4′-phosphopantetheinyl transferase) for post-translational modification of ACP by converting inactive ACP to functional ACP. The improved production of EPA and DHA was reported by endogenous PPTase activity in *Aurantiochytrium* by coexpressing the *pfaE* gene from *S*. *japonica* strain KCTC 22435 [86]. This illustrates the new strategy for improved DHA production in Thraustochytrids. Overall, BLAST similarity search of all the individual *pfa* genes of PUFA synthase complex revealed its homology with *S*. *baltica* strain 128 with 100% similarity score at amino acid level.

Structure predictions of *Shewanella* sp. N2AIL with Phyre2 and the closely related PDB structures of individual domains as templates support the identity of the individual *pfa* domains (Figure 8). The homology-based structure of the *PfaA*-KS domain of *Shewanella* sp. N2AIL was found to have a thiolase fold, which is the characteristic of a typical KS domain [87]. The MAT domain structure was constructed based on the crystal structure of MCAT of *S*. *pneumonia* with a sequence identity of 33% and showed an RMSD value of 0.021% [88] by having two subdomains of αβ connected by two linkers. The smaller subdomain consists of four antiparallel β sheets covered by 2 α helices similar to *S*. *pneumonia*, while the larger subdomain contained 2 antiparallel β strands shifted outwards and surrounded by 12 α-helices unlike 6 antiparallel β strands surrounded by 11 α-helices of the template. The structure of ACP of *Shewenella* sp. N2AIL was constructed based on *Thermus thermophilus* Hb8 with 35.72 % sequence identity. It consists of five repetitions of four well folded right turn α-helices with the arrangement of helices 1, 2, 4 in up-down-down topology, similar to the solution structure of ACP from *Mycobacterium tuberculosis* [89]. The Rossman folded KR domain is composed of six parallel beta sheets flanked by structural and catalytic domains at the N and C terminal, respectively, having five α-helices each, as also reported in the type-1 polyketide synthase reductases of *Moritella marina* with seven strands β-sheet flanked by eight α-helices [90]. The DH domain is represented as a hotdog fold with seven antiparallel β sheets on both the N and C terminal and two hotdog α helices acting as a sausage between them, similar to the DH domain of the CurF module of Curacin polyketide synthase [91]. 

## 4. Conclusions

A genome study of *Shewanella* sp. N2AIL has been characterized by the presence of genes for important secondary metabolites and for the reduction of heavy metals such as iron, copper, selenite, nitrate, iodine, and arsenic. The genome analysis also indicated plenty of other stress-responsive genes related to pH, temperature, salt, osmotic and oxidative stresses. This study emphasized mainly genes related to iron metabolisms such as *mtr* gene cluster, TonB dependant siderophore receptor, *feo* and *fur* genes for iron uptake and its regulation, *bolA* gene linked with biofilm formation and bioelectricity generation, and most importantly, PUFA biosynthetic gene cluster (*PfaA-D* and *PfaE*) for EPA production in *Shewanella* sp. N2AIL genome. The presence of these genes in the genome expands its potential to be developed as an industrially important strain that could be utilized as a model organism to study heavy metal adaptation and PUFA biosynthesis. 

## Figures and Tables

**Table 1:** Secondary metabolite gene cluster of *Shewanella* sp. N2AIL genome.

Secondary Metabolite	Total Length (ntd.)	Location	Core Biosynthetic Gene Cluster
PUFA	56,306	ctg1_236 ctg1_238	PUFAKS hgIE
Betalactone	29,577	ctg3_15 ctg3_21	AMP binding HMGL-like
RiPP like	10,900	ctg33_14	DUF692
Siderophore	11,926	ctg15_37	lucA_lucC
Arylpolyene	28,285	ctg16_6 ctg16_9	APE_KS1 APE_KS2

**Table 2:** Antibiotic resistant gene prediction of *Shewanella* sp. N2AIL genome.

Antibiotic Class	Gene/Protein	Identity (%)	Resistance Mechanism	Function	Location
Fluoroquinolone, Tetracycline	adeF	65.03	Antibiotic efflux	AdeF, a membrane fusion protein of multuidrug efflux system AdeFGH	NODE_8_length_146061_cov_52.115607_51718_48584
Fluoroquinolone, Tetracycline	adeF	42.43	Antibiotic efflux	AdeF, a membrane fusion protein of multuidrug efflux system AdeFGH	NODE_2_length_334757_cov_47.438374_143168_146326
Elfamycin	EF-Tu mutants	87.53	Antibiotic target alteration	*Escherichia coli* elongation factor Tu that confer resistance to drug class elfamycin	NODE_40_length_41192_cov_51.690807_25364_24180
Fluoroquinolone, Diaminopyrimidine, phenicol Carbapenem, Cephalosporin, Penam	RsmA OXA-551	86.67 97.97	Antibiotic efflux Antibiotic inactivation	RsmA confer resistance by negatively regulating MexEF-OprN overexpression and thus virulence of *P. aeruginosa*. OXA enzymes confer resistance to Carbapenem particularly in *Acinetobacter baumannii*	NODE_2_length_334757_cov_47.438374_217007_217204 NODE_32_length_54861_cov_49.238334_7238_6351

**Table 3:** Genes associated with stress responses in *Shewanella* sp. N2AIL genome.

Class	Gene/Protein Name	Function
Temperature stress	*rpoE*	Encodes RNA polymerase sigma factor σ24 necessary for growth at high and low temperature
	*degQ*	A RpoE dependant periplasmic serine protease required for bacterial growth at high temperature
	Csp family proteins (4No.)	Serve as RNA chaperone to regulate transcription, translation and degradation of mRNA
	Hsp family proteins (6No.)	Heat shock proteins have key role in protein folding, assembly, degradation and their transport across membranes
Oxidative stress	*chrR*	Transcription activator which is a part of repE2 operon and senses ROS
	Glutathione peroxidase	A RpoE dependant Glutathione peroxidase cope with oxidative stress
	Thioredoxin reductase	Essential protein for regulating redox balance in cell
	Glutathione reductase	Role in cellular control of Reactive oxygen species thus maintaining balance in cell
	*katG*, *katE*	Genes involved in antioxidant defence against H_2_O_2_ induced stress
Heavy metal stress		
Iron	*mtrBAC*, *mtrFED*	Encoding outer membrane cytochrome C responsible for iron reduction
	*feoA*, *feoB*	A direct iron transport complex for uptake of soluble form of iron- Fe2^+^
	Ton B receptor proteins	A chelator for transport of insoluble form of iron-Fe3^+^ using TonB receptor present on outer membrane.
	*fur*	A ferric uptake regulation protein required for Iron homeostasis
Selenite	*fccA*	Periplasmic fumarate reductase
	*cymA*	Membrane bound Type C Cytochrome assist FccA for reduction of selenite.
Copper	*cusA*	A heavy metal efflux RND transporter protein for transport of copper/silver across membranes.
	*copA*	Copper sensory histidine kinase for its role in copper stress resistance.
Nitrate	*nar(narQ*, *narP)*	A membrane bound two component system for regulation of Nap operon
	*nap(napDEF)*	Periplasmic nitrate reductase gene for reduction and excretion of nitrate reducing end products out of cell.
Iodine	*mtrAB*	Periplasmic cytochrome c protein mtrAand Outer membrane anchored mtrB required for iodate reduction
Arsenic	*acr3*, *acrR*	Arsenic resistant proteins
Salt stress	*kdpD*, *kdpE* *trkH*	A transporter for potassium uptake helps bacteria in adaptation to salt stress
	*rpoS*	A sigma factor regulatory protein
	*betA*, *betB*	Betaine aldehyde dehydrogenase and Choline dehydrogenase are required for betaine and choline uptake by cell
	-	Encoding Glutamate-5 kinase enzyme for proline biosynthesis
pH stress		
Acid stress	*rpoS*	A sigma factor σ38 or a global regulatory protein
	*csgB*, *csgEFG*	Genes encodes for cell envelope structure or curli assembly
	*pstA*, *pstB*	Phosphate ABC transporter permease protein in response to acid stress
	*phoB*, *phoU*	Phosphate transport regulatory protein
Alkaline stress	*nhaA*, *nhaR*	A membrane bound Na^+^/H^+^ antiporter system responsible for adaptation to alkaline stress
	*cysP*, *cysT*, *cysW*, *cysA*, *cysB*	Sulfate ABC transporter proteins
Osmotic stress	Two component *envZ*/*ompR* system	Osmolarity sensory histidine kinase to mediate osmotic stress response in bacteria
	*lysR*	LysR family transcription regulator

## Data Availability

The data presented in this study is available in NCBI database under accession numbers JALBYT000000000 and OM919690 for draft genome sequence and 16S rRNA gene sequence, respectively.

## Supplementary Materials

The following supporting information can be downloaded at: https://www.mdpi.com/article/10.3390/biology11050632/s1, Figure S1 Subsystem distribution of genes in various categories in *Shewanella* sp. N2AIL genome; Table S1: OrthoANI values calculated from the OAT software; Table S2: Pairwise *in silico* DNA–DNA hybridization (DDH) scores of *Shewanella* sp. N2AIL and closely related type strains.

## References

[1] Shewan J.M., Hobbs G., Hodgkiss W. (1960). A determinative scheme for the identification of certain genera of Gram-negative bacteria, with special reference to the Pseudomonadaceae. J. Appl. Bacteriol..

[2] Hammer B., Long H. (1941). Factors influencing bacterial growth in butter. Bacteriol. Rev..

[3] Lee J., Gibson D., Shewan J.M. (1977). A numerical taxonomic study of some Pseudomonas-like marine bacteria. Microbiology.

[4] MacDonell M., Colwell R. (1985). Phylogeny of the Vibrionaceae, and recommendation for two new genera, Listonella and Shewanella. Syst. Appl. Microbiol..

[5] Kim J.-Y., Yoo H.-S., Lee D.-H., Park S.-H., Kim Y.-J., Oh D.-C. (2016). *Shewanella algicola* sp. nov., a marine bacterium isolated from brown algae. Int. J. Syst. Evol. Microbiol..

[6] Luhtanen A.-M., Eronen-Rasimus E., Kaartokallio H., Rintala J.-M., Autio R., Roine E. (2014). Isolation and characterization of phage–host systems from the Baltic Sea ice. Extremophiles.

[7] Li Z., Lin S., Liu X., Tan J., Pan J., Yang H. (2014). A freshwater bacterial strain, Shewanella sp. Lzh-2, isolated from Lake Taihu and its two algicidal active substances, hexahydropyrrolo [1, 2-a] pyrazine-1, 4-dione and 2, 3-indolinedione. Appl. Microbiol. Biotechnol..

[8] Wang M.-Q., Sun L. (2016). *Shewanella inventionis* sp. nov., isolated from deep-sea sediment. Int. J. Syst. Evol. Microbiol..

[9] Nichols D.S., Nichols P.D., Russell N.J., Davies N.W., McMeekin T.A. (1997). Polyunsaturated fatty acids in the psychrophilic bacterium *Shewanella gelidimarina* ACAM 456T: Molecular species analysis of major phospholipids and biosynthesis of eicosapentaenoic acid. Biochim. Biophys. Acta-Lipids Lipid Metab..

[10] Valentine R.C., Valentine D.L. (2004). Omega-3 fatty acids in cellular membranes: A unified concept. Prog. Lipid Res..

[11] Gao H., Obraztova A., Stewart N., Popa R., Fredrickson J.K., Tiedje J.M., Nealson K.H., Zhou J. (2006). *Shewanella loihica* sp. nov., isolated from iron-rich microbial mats in the Pacific Ocean. Int. J. Syst. Evol. Microbiol..

[12] Beblawy S., Bursac T., Paquete C., Louro R., Clarke T.A., Gescher J. (2018). Extracellular reduction of solid electron acceptors by *Shewanella oneidensis*. Mol. Microbiol..

[13] Fredrickson J.K., Romine M.F., Beliaev A.S., Auchtung J.M., Driscoll M.E., Gardner T.S., Nealson K.H., Osterman A.L., Pinchuk G., Reed J.L. (2008). Towards environmental systems biology of Shewanella. Nat. Rev. Microbiol..

[14] Jiang S., Ho C.T., Lee J.-H., Van Duong H., Han S., Hur H.-G. (2012). Mercury capture into biogenic amorphous selenium nanospheres produced by mercury resistant *Shewanella putrefaciens* 200. Chemosphere.

[15] Suganthi S.H., Murshid S., Sriram S., Ramani K. (2018). Enhanced biodegradation of hydrocarbons in petroleum tank bottom oil sludge and characterization of biocatalysts and biosurfactants. J. Environ. Manag..

[16] Lemaire O.N., Honoré F.A., Tempel S., Fortier E.M., Leimkühler S., Méjean V., Iobbi-Nivol C. (2019). *Shewanella decolorationis* LDS1 chromate resistance. Appl. Environ. Microbiol..

[17] de Santana F.S., Gracioso L.H., Karolski B., dos Passos Galluzzi Baltazar M., Mendes M.A., do Nascimento C.A.O., Perpetuo E.A. (2019). Isolation of bisphenol A-tolerating/degrading *Shewanella haliotis* strain MH137742 from an estuarine environment. Appl. Biochem. Biotechnol..

[18] Zou L., Huang Y.-h., Long Z.-e., Qiao Y. (2019). On-going applications of *Shewanella* species in microbial electrochemical system for bioenergy, bioremediation and biosensing. World J. Microbiol. Biotechnol..

[19] Vaidya S., Dev K., Sourirajan A. (2018). Distinct osmoadaptation strategies in the strict halophilic and halotolerant bacteria isolated from Lunsu salt water body of North West Himalayas. Curr. Microbiol..

[20] Roeßler M., Müller V. (2001). Osmoadaptation in bacteria and archaea: Common principles and differences. Environ. Microbiol..

[21] Hau H.H., Gralnick J.A. (2007). Ecology and biotechnology of the genus Shewanella. Annu. Rev. Microbiol..

[22] Gram L., Trolle G., Huss H.H. (1987). Detection of specific spoilage bacteria from fish stored at low (0 C) and high (20 C) temperatures. Int. J. Food Microbiol..

[23] Yagi H., Fujise A., Itabashi N., Ohshiro T. (2018). Characterization of a novel endo-type alginate lyase derived from *Shewanella* sp. YH1. J. Biochem..

[24] Frolova G., Pavel K., Shparteeva A., Nedashkovskaya O., Gorshkova N., Ivanova E., Mikhailov V. (2005). Lipid composition of novel *Shewanella* species isolated from Far Eastern seas. Microbiology.

[25] Russell N.J., Nichols D.S. (1999). Polyunsaturated fatty acids in marine bacteria—a dogma rewritten. Microbiology.

[26] Kawamoto J., Kurihara T., Yamamoto K., Nagayasu M., Tani Y., Mihara H., Hosokawa M., Baba T., Sato S.B., Esaki N. (2009). Eicosapentaenoic acid plays a beneficial role in membrane organization and cell division of a cold-adapted bacterium, *Shewanella livingstonensis* Ac10. J. Bacteriol..

[27] Fang J., Chan O., Kato C., Sato T., Peeples T., Nigggemeyer K. (2003). Phospholipid FA of piezophilic bacteria from the deep sea. Lipids.

[28] Wang F., Wang J., Jian H., Zhang B., Li S., Wang F., Zeng X., Gao L., Bartlett D.H., Yu J. (2008). Environmental adaptation: Genomic analysis of the piezotolerant and psychrotolerant deep-sea iron reducing bacterium *Shewanella piezotolerans* WP3. PLoS ONE.

[29] Wang F., Xiao X., Ou H.-Y., Gai Y., Wang F. (2009). Role and regulation of fatty acid biosynthesis in the response of *Shewanella piezotolerans* WP3 to different temperatures and pressures. J. Bacteriol..

[30] Horvath A., Koletzko B., Szajewska H. (2007). Effect of supplementation of women in high-risk pregnancies with long-chain polyunsaturated fatty acids on pregnancy outcomes and growth measures at birth: A meta-analysis of randomized controlled trials. Br. J. Nutr..

[31] Bang H., Dyerberg J., Hjørne N. (1976). The composition of food consumed by Greenland Eskimos. Acta Med. Scand..

[32] Lagarde M., Burtin M., Sprecher H., Dechavanne M., Renaud S. (1983). Potentiating effect of 5, 8, 11-eicosatrienoic acid on human platelet aggregation. Lipids.

[33] Das U.N. (2013). Autism as a disorder of deficiency of brain-derived neurotrophic factor and altered metabolism of polyunsaturated fatty acids. Nutrition.

[34] Konstantinidis K.T., Serres M.H., Romine M.F., Rodrigues J.L., Auchtung J., McCue L.-A., Lipton M.S., Obraztsova A., Giometti C.S., Nealson K.H. (2009). Comparative systems biology across an evolutionary gradient within the *Shewanella* genus. Proc. Natl. Acad. Sci. USA.

[35] Weisburg W.G., Barns S.M., Pelletier D.A., Lane D.J. (1991). 16S ribosomal DNA amplification for phylogenetic study. J. Bacteriol..

[36] Dos Santos H.R.M., Argolo C.S., Argôlo-Filho R.C., Loguercio L.L. (2019). A 16S rDNA PCR-based theoretical to actual delta approach on culturable mock communities revealed severe losses of diversity information. BMC Microbiol..

[37] Sanger F., Nicklen S., Coulson A.R. (1977). DNA sequencing with chain-terminating inhibitors. Proc. Natl. Acad. Sci. USA.

[38] Tamura K., Stecher G., Kumar S. (2021). MEGA11: Molecular evolutionary genetics analysis version 11. Mol. Biol. Evol..

[39] Yoon S.-H., Ha S.-M., Lim J., Kwon S., Chun J. (2017). A large-scale evaluation of algorithms to calculate average nucleotide identity. Antonie Van Leeuwenhoek.

[40] Bertels F., Silander O.K., Pachkov M., Rainey P.B., Van Nimwegen E. (2014). Automated reconstruction of whole-genome phylogenies from short-sequence reads. Mol. Biol. Evol..

[41] Aziz R.K., Bartels D., Best A.A., DeJongh M., Disz T., Edwards R.A., Formsma K., Gerdes S., Glass E.M., Kubal M. (2008). The RAST Server: Rapid annotations using subsystems technology. BMC Genom..

[42] Grissa I., Vergnaud G., Pourcel C. (2007). CRISPRFinder: A web tool to identify clustered regularly interspaced short palindromic repeats. Nucleic Acids Res..

[43] McArthur A.G., Waglechner N., Nizam F., Yan A., Azad M.A., Baylay A.J., Bhullar K., Canova M.J., De Pascale G., Ejim L. (2013). The comprehensive antibiotic resistance database. Antimicrob. Agents Chemother..

[44] Medema M.H., Blin K., Cimermancic P., De Jager V., Zakrzewski P., Fischbach M.A., Weber T., Takano E., Breitling R. (2011). antiSMASH: Rapid identification, annotation and analysis of secondary metabolite biosynthesis gene clusters in bacterial and fungal genome sequences. Nucleic Acids Res..

[45] Wu S., Zhu Z., Fu L., Niu B., Li W. (2011). WebMGA: A customizable web server for fast metagenomic sequence analysis. BMC Genom..

[46] Darling A.C., Mau B., Blattner F.R., Perna N.T. (2004). Mauve: Multiple alignment of conserved genomic sequence with rearrangements. Genome Res..

[47] Seemann T. (2014). Prokka: Rapid prokaryotic genome annotation. Bioinformatics.

[48] Chen T., Zhang H., Liu Y., Liu Y.-X., Huang L. (2021). EVenn: Easy to create repeatable and editable Venn diagrams and Venn networks online. J. Genet. Genom..

[49] Schultz J., Copley R.R., Doerks T., Ponting C.P., Bork P. (2000). SMART: A web-based tool for the study of genetically mobile domains. Nucleic Acids Res..

[50] Kelley L.A., Sternberg M.J. (2009). Protein structure prediction on the Web: A case study using the Phyre server. Nat. Protoc..

[51] Singh A., Kaushik R., Mishra A., Shanker A., Jayaram B. (2016). ProTSAV: A protein tertiary structure analysis and validation server. Biochim. Biophys. Acta-Proteins Proteom..

[52] Richter M., Rosselló-Móra R. (2009). Shifting the genomic gold standard for the prokaryotic species definition. Proc. Natl. Acad. Sci. USA.

[53] Konstantinidis K.T., Tiedje J.M. (2005). Genomic insights that advance the species definition for prokaryotes. Proc. Natl. Acad. Sci. USA.

[54] Li J., Yu H., Yang X., Dong R., Liu Z., Zeng M. (2020). Complete genome sequence provides insights into the quorum sensing-related spoilage potential of *Shewanella baltica* 128 isolated from spoiled shrimp. Genomics.

[55] Tseng S.-Y., Tung K.-C., Cheng J.-F., Lee Y.-H., Wu Z.-Y., Hong Y.-K., Chen S.-Y., Huang Y.-T., Liu P.-Y. (2018). Genome characterization of bile-isolated *Shewanella algae* ACCC. Gut Pathog..

[56] Li Z., Song F., Chen M. (2021). Complete Genome Sequence of *Shewanella* sp. Strain Lzh-2, an Algicidal Bacterial Strain Isolated from Lake Taihu, People’s Republic of China. Microbiol. Resour. Announc..

[57] Cimermancic P., Medema M.H., Claesen J., Kurita K., Brown L.C.W., Mavrommatis K., Pati A., Godfrey P.A., Koehrsen M., Clardy J. (2014). Insights into secondary metabolism from a global analysis of prokaryotic biosynthetic gene clusters. Cell.

[58] Letzel A.-C., Pidot S.J., Hertweck C. (2014). Genome mining for ribosomally synthesized and post-translationally modified peptides (RiPPs) in anaerobic bacteria. BMC Genom..

[59] Chen R., Wong H.L., Kindler G.S., MacLeod F.I., Benaud N., Ferrari B.C., Burns B.P. (2020). Discovery of an abundance of biosynthetic gene clusters in shark bay microbial mats. Front. Microbiol..

[60] Huang Y.-T., Tang Y.-Y., Cheng J.-F., Wu Z.-Y., Mao Y.-C., Liu P.-Y. (2018). Genome analysis of multidrug-resistant *Shewanella algae* isolated from human soft tissue sample. Front. Pharmacol..

[61] Zhang X., Ruan Y., Liu W., Chen Q., Gu L., Guo A. (2021). Whole genome sequencing and genome annotation of Dermacoccus abyssi strain HZAU 226 isolated from spoiled eggs. Genomics.

[62] Fujii S., Nakasone K., Horikoshi K. (1999). Cloning of two cold shock genes, cspA and cspG, from the deep-sea psychrophilic bacterium *Shewanella violacea* strain DSS12. FEMS Microbiol. Lett..

[63] Dai J., Wei H., Tian C., Damron F.H., Zhou J., Qiu D. (2015). An extracytoplasmic function sigma factor-dependent periplasmic glutathione peroxidase is involved in oxidative stress response of *Shewanella oneidensis*. BMC Microbiol..

[64] Yuan J., Wei B., Shi M., Gao H. (2011). Functional assessment of EnvZ/OmpR two-component system in *Shewanella oneidensis*. PLoS ONE.

[65] Fu X., Wang D., Yin X., Du P., Kan B. (2014). Time course transcriptome changes in *Shewanella algae* in response to salt stress. PLoS ONE.

[66] Leaphart A.B., Thompson D.K., Huang K., Alm E., Wan X.-F., Arkin A., Brown S.D., Wu L., Yan T., Liu X. (2006). Transcriptome profiling of *Shewanella oneidensis* gene expression following exposure to acidic and alkaline pH. J. Bacteriol..

[67] Toes A.-C.M., Daleke M.H., Kuenen J.G., Muyzer G. (2008). Expression of copA and cusA in *Shewanella* during copper stress. Microbiology.

[68] Li X.-G., Zhang W.-J., Xiao X., Jian H.-H., Jiang T., Tang H.-Z., Qi X.-Q., Wu L.-F. (2018). Pressure-regulated gene expression and enzymatic activity of the two periplasmic nitrate reductases in the deep-sea bacterium *Shewanella piezotolerans* WP3. Front. Microbiol..

[69] Li D.-B., Cheng Y.-Y., Wu C., Li W.-W., Li N., Yang Z.-C., Tong Z.-H., Yu H.-Q. (2014). Selenite reduction by *Shewanella oneidensis* MR-1 is mediated by fumarate reductase in periplasm. Sci. Rep..

[70] Shi L., Chen B., Wang Z., Elias D.A., Mayer M.U., Gorby Y.A., Ni S., Lower B.H., Kennedy D.W., Wunschel D.S. (2006). Isolation of a high-affinity functional protein complex between OmcA and MtrC: Two outer membrane decaheme c-type cytochromes of *Shewanella oneidensis* MR-1. J. Bacteriol..

[71] Coursolle D., Gralnick J.A. (2012). Reconstruction of extracellular respiratory pathways for iron (III) reduction in *Shewanella oneidensis* strain MR-1. Front. Microbiol..

[72] Yang Y., Chen J., Qiu D., Zhou J. (2013). Roles of UndA and MtrC of *Shewanella*
*putrefaciens* W3-18-1 in iron reduction. BMC Microbiol..

[73] Dong Z., Guo S., Fu H., Gao H. (2017). Investigation of a spontaneous mutant reveals novel features of iron uptake in *Shewanella oneidensis*. Sci. Rep..

[74] Liu L., Li S., Wang S., Dong Z., Gao H. (2018). Complex iron uptake by the putrebactin-mediated and Feo systems in *Shewanella oneidensis*. Appl. Environ. Microbiol..

[75] Yang X.-W., He Y., Xu J., Xiao X., Wang F.-P. (2013). The regulatory role of ferric uptake regulator (Fur) during anaerobic respiration of *Shewanella piezotolerans* WP3. PLoS ONE.

[76] Aldea M., Hernandez-Chico C., De la Campa A., Kushner S., Vicente M. (1988). Identification, cloning, and expression of bolA, an ftsZ-dependent morphogene of Escherichia coli. J. Bacteriol..

[77] Dressaire C., Moreira R.N., Barahona S., Alves de Matos A.P., Arraiano C.M. (2015). BolA is a transcriptional switch that turns off motility and turns on biofilm development. MBio.

[78] Silva A.V., Edel M., Gescher J., Paquete C.M. (2020). Exploring the effects of bolA in biofilm formation and current generation by *Shewanella oneidensis* MR-1. Front. Microbiol..

[79] Okuyama H., Orikasa Y., Nishida T., Watanabe K., Morita N. (2007). Bacterial genes responsible for the biosynthesis of eicosapentaenoic and docosahexaenoic acids and their heterologous expression. Appl. Environ. Microbiol..

[80] Jiang H., Zirkle R., Metz J.G., Braun L., Richter L., Van Lanen S.G., Shen B. (2008). The role of tandem acyl carrier protein domains in polyunsaturated fatty acid biosynthesis. J. Am. Chem. Soc..

[81] Hayashi S., Satoh Y., Ujihara T., Takata Y., Dairi T. (2016). Enhanced production of polyunsaturated fatty acids by enzyme engineering of tandem acyl carrier proteins. Sci. Rep..

[82] Shulse C.N., Allen E.E. (2011). Widespread occurrence of secondary lipid biosynthesis potential in microbial lineages. PLoS ONE.

[83] Orikasa Y., Tanaka M., Sugihara S., Hori R., Nishida T., Ueno A., Morita N., Yano Y., Yamamoto K., Shibahara A. (2009). pfaB products determine the molecular species produced in bacterial polyunsaturated fatty acid biosynthesis. FEMS Microbiol. Lett..

[84] Li Z., Meng T., Ling X., Li J., Zheng C., Shi Y., Chen Z., Li Z., Li Q., Lu Y. (2018). Overexpression of malonyl-CoA: ACP transacylase in Schizochytrium sp. to improve polyunsaturated fatty acid production. J. Agric. Food Chem..

[85] Pasta S., Witkowski A., Joshi A.K., Smith S. (2007). Catalytic residues are shared between two pseudosubunits of the dehydratase domain of the animal fatty acid synthase. Chem. Biol..

[86] Wang S., Lan C., Wang Z., Wan W., Cui Q., Song X. (2020). PUFA-synthase-specific PPTase enhanced the polyunsaturated fatty acid biosynthesis via the polyketide synthase pathway in Aurantiochytrium. Biotechnol. Biofuels.

[87] Nanson J.D., Himiari Z., Swarbrick C., Forwood J.K. (2015). Structural characterisation of the beta-ketoacyl-acyl carrier protein synthases, FabF and FabH, of Yersinia pestis. Sci. Rep..

[88] Hong S.K., Kim K.H., Park J.K., Jeong K.-W., Kim Y., Kim E.E. (2010). New design platform for malonyl-CoA-acyl carrier protein transacylase. FEBS Lett..

[89] Wong H.C., Liu G., Zhang Y.-M., Rock C.O., Zheng J. (2002). The solution structure of acyl carrier protein from Mycobacterium tuberculosis. J. Biol. Chem..

[90] Santín O., Moncalián G. (2018). Loading of malonyl-CoA onto tandem acyl carrier protein domains of polyunsaturated fatty acid synthases. J. Biol. Chem..

[91] Dillon S.C., Bateman A. (2004). The Hotdog fold: Wrapping up a superfamily of thioesterases and dehydratases. BMC Bioinform..

