# Supplementary material for: Genomic Insights into Omega-3 Polyunsaturated Fatty Acid Producing Shewanella sp. N2AIL from Fish Gut
Source: Biology (Basel). 2022 Apr 21;11(5):632. doi: 10.3390/biology11050632 (PMC9138089; doi:10.3390/biology11050632)
Supplement: Supplementary file 1 [file biology-11-00632-s001.zip › Supplementary Figure S1.pdf]

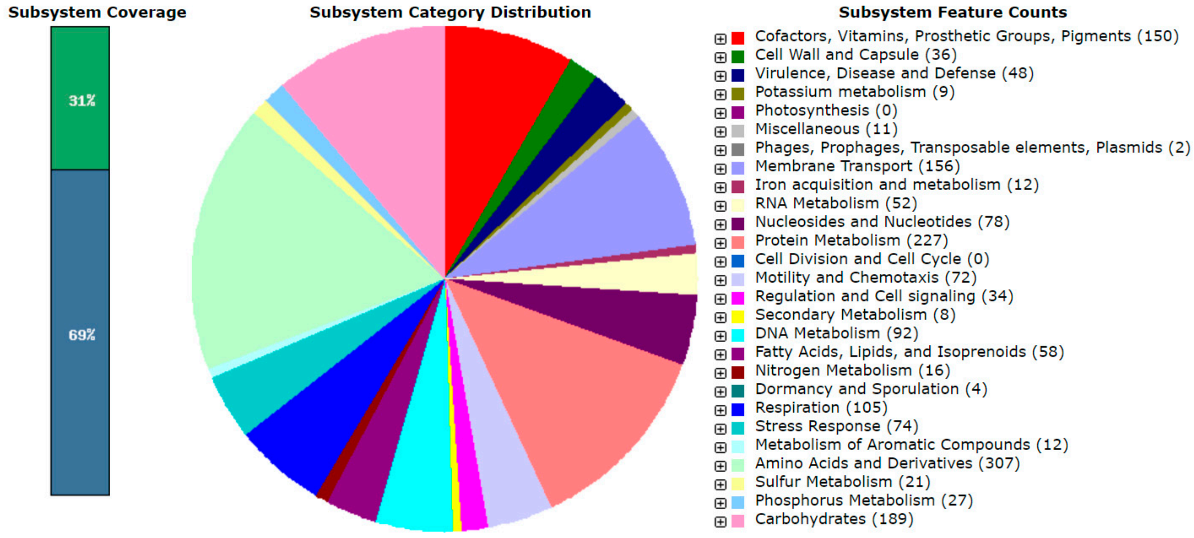

Supplementary Figure S1 Subsystem distribution of genes in various categories in *Shewanella* sp. N2AIL genome.
